# Cerebrospinal fluid hypovolemia syndrome with benign course

**DOI:** 10.4103/0972-2327.74202

**Published:** 2010

**Authors:** K. N. Ramesha, Kesavadas Chandrashekaran, Sanjeev V. Thomas

**Affiliations:** Department of Neurology, Sree Chitra Tirunal Institute for Medical Sciences and Technology, Trivandrum 695 011, India; Department of Imaging Sciences & Interventional Radiology, Sree Chitra Tirunal Institute for Medical Sciences and Technology, Trivandrum 695 011, India

**Keywords:** Headache, spontaneous intracranial hypotension, venous distension sign

## Abstract

**Background:**

The cerebrospinal fluid hypovolemia syndrome (CHS) is an under recognized cause of headache. This study was designed to highlight the clinico-radiological and cerebrospinal fluid (CSF) picture of CHS and their long-term outcome from a tertiary referral center.

**Materials and Methods:**

The CHS was diagnosed on the basis of the criteria proposed by Chung *et al*. Cases with CSF rhinorrhoea or other CSF leak or head trauma were excluded from the study.

**Results:**

The study included eight consecutive cases of CHS diagnosed over the past 7 years from 2001. The mean age at diagnosis was 40.7 years (range, 34-56 years) and male-to-female ratio was 1:3. All patients presented with orthostatic headache of subacute onset and normal neurological examination. Magnetic resonance imaging studies of all patients showed hyperintensity of pachymeninges in T2W sequences, venous distension sign, and diffuse pachymeningeal gadolinium enhancement. The descent of the brainstem and subdural effusion were noted in two each (25%). CSF study (n = 5) showed low opening pressure in three (60%), and mild pleocytosis with elevated protein in two each (40%). The mean time to complete recovery with conservative treatment alone was 25.6 days. All radiological signs disappeared with clinical improvement in three patients where follow-up imaging was done. On mean follow-up period of 3.6 years, all were asymptomatic without any recurrence of CHS.

**Conclusion:**

CHS can resolve completely with conservative management and intervention with subdural blood patch or surgical repair would be required only if symptoms persist for more than 1 month.

## Introduction

The cerebrospinal fluid hypovolemia syndrome (CHS) or spontaneous intracranial hypotension (SIH) is characterized by orthostatic headache, low CSF pressure, and diffuse pachymeningeal gadolinium enhancement in the magnetic resonance imaging (MRI).[1–4] A dural tear or spinal meningeal diverticula are the suggested underlying pathogenic mechanisms.[1,3] It is often under recognized or misdiagnosed.[1] The lack of awareness of this condition may lead to unnecessary investigations, delayed diagnosis, and related morbidity. This is the first report of long-term follow-up and outcome of CHS from India. We report eight consecutive cases of CHS diagnosed at our center with their outcome on long-term follow-up.

## Materials and Methods

This retrospective study was conducted at a tertiary care university hospital for neurological and cardiovascular diseases in South India. We identified patients with CHS by screening the medical records of all patients who had presented with headache (*n* = 2932) to the neurology outpatient service during the period 2001-2007. We diagnosed CHS by the criteria recommended by Chung *et al*.[1] which requires the presence of two of the three features, namely orthostatic headache, low CSF pressure (<60 mm of water), and diffuse pachymeningeal gadolinium enhancement in the MRI were satisfied. The orthostatic headache[5] was defined as a headache that occurs in <15 min after assuming the upright posture and disappears or improves in <30 min after assuming the recumbent position. MRI was done prior to the CSF study in all the patients. The presence of a convex inferior border of the dominant transverse sinus in the nonenhanced sagittal T1W image was classified as venous distension sign (VDS) in the MRI.[6] Neuroinfections were ruled out by CSF analysis. Cases with history of recent CSF rhinorrhoea or otorrhoea, head trauma, and lumbar puncture were excluded from the study. All patients who were included were contacted about their health status at last follow-up either by mail or by telephone using a structured proforma.

## Results

We identified eight cases of CHS from 2932 cases of headache that were screened for the diagnosis in this Institute during the period 2001-2007. The clinical features and outcome of all patients are summarized in Table 1.

**Figure 1:**
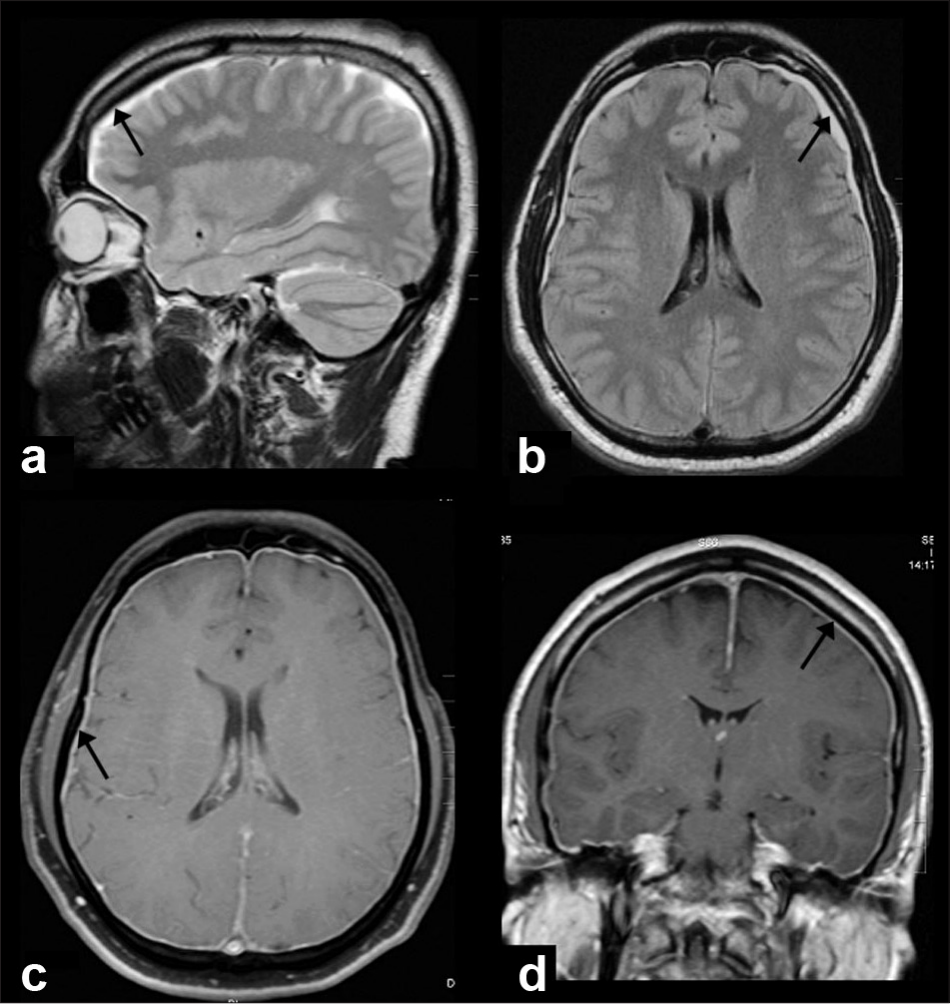
Sagittal T2 weighted (a) and axial FLAIR (b) images show uniform hyperintensity of pachymeninges (arrows). Axial fat suppressed postcontrast (c) and coronal postcontrast (d) images show diffuse pachymeningeal gadolinium enhancement

**Figure 2:**
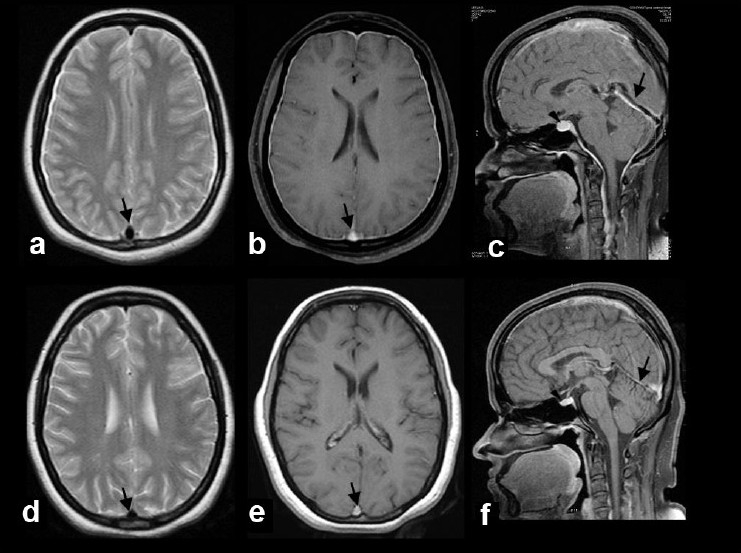
Axial T2 weighted (a), postcontrast axial (b), and postcontrast sagittal (c) images reveal distension of the sagittal sinus, straight sinus (arrows), and enlarged pituitary gland (arrow head) due to hyperemia. Axial T2 weighted (d), postcontrast axial (e), and postcontrast sagittal (f) images of a repeat MRI done after 3 months shows resolution of pachymeningeal T2 hyperintensity, gadolinium enhancement, pituitary enlargement, and venous distension

**Figure 3:**
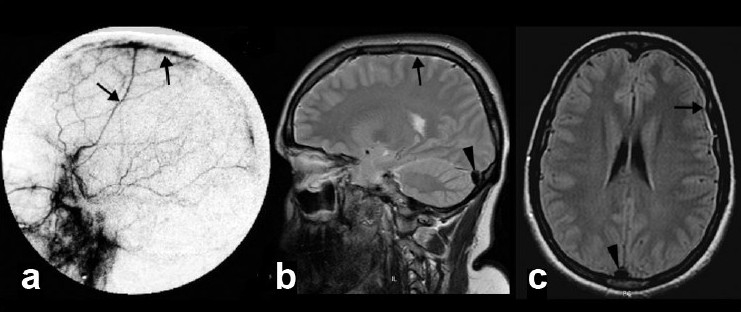
Lateral view of digital subtraction angiography of left carotid angiogram (a) shows increased vascularity and early filling of the pachymeninges covering the sagittal sinus through meningeal arteries (arrow). This appearance is due to meningeal vasodilatation. Sagittal T2 weighted (b) and axial FLAIR (c) images show pachymeningeal T2 hyperintensity (black arrow) and venous distension sign (black arrowhead)

The mean age at diagnosis was 40.7 years (range, 34-56 years) and male-to-female ratio was 1:3. The mean interval between the onset of symptoms and admission to the hospital was 17.6 days (range, 1-33 days). All had orthostatic headache of throbbing to dull aching type, mainly in the occipital region and nausea. None had other types of headache at presentation. In addition, one patient had binocular diplopia without any obvious cranial nerve palsy. The other symptoms included neck pain, tinnitus, fullness in the ear, vertigo, and vomiting. Neurological examination was normal in all patients. MRI studies showed hyperintensity of pachymeninges in T2W and fluid attenuated inversion recovery (FLAIR) sequences and VDS with diffuse pachymeningeal gadolinium enhancement in all eight cases (100%) [Figure 1]. The MRI findings are summarized in Table 2. In addition, there was subdural effusion and descent of the brainstem with flattening of the pons in two patients (25%). One patient showed pituitary hyperemia (12.5%). The follow-up MRI done in three patients revealed resolution of all MRI findings including the VDS sign [Figure 2]. Digital subtraction angiogram (DSA) in one patient showed early venous filling [Figure 3].

**Table 1:** Clinical features, CSF picture, and outcome of study cohort

Age/sex	Symptoms	CSF pressure (mm of water)	CSF cell count	CSF protein (mg%)	Past history	Time to full recovery (days)	Follow-up (years)	Status at lastfollow-up
34/F	OH, T, V, FE	ND	ND	ND	Nil	30	2.4	A
39/F	OH,	40	2	40	Nil	10	2.6	A
34/M	OH, BD	40	20	115	MHT	30	1.2	A
38/F	OH, V	ND	ND	ND	SA	21	2.1	VH
45/F	OH	70	5	34	Nil	24	2.3	A
36/F	OH, T, V	30	2	50	Nil	30	4	A
56/F	OH, V, NP	D	D	D	SA	12	7.7	A
44/M	OH	60	10	15	Nil	11	7.2	VH

M, Male; F, Female; OH, Orthostatic headache; T, Tinnitus; NP, Neck pain; FE, Fullness in the ear; V, Vomiting; BD, Binocular diplopia; ND, Not done; D, Dry tap; MHT, Nonspecific mild head trauma without CSF leak in the past; SA, Spinal anesthesia many years back; A, Asymptomatic; VH, Vascular headache.

**Table 2:** Shows magnetic resonance imaging findings of study cohort

Pachymeningeal hyperintensity in T2W	Pachymeningeal hyperintensity in FLAIR	Diffuse pachymeningeal gadolinium enhancement	VDS signs	Subdural effusion	Pituitary hyperemia	Brainstem descent
+	+	+	+	-	-	+
+	+	+	+	-	-	-
+	+	+	+	+	-	-
+	+	+	+	-	-	-
+	+	+	+	-	-	-
+	+	+	+	+	-	-
+	+	+	+	-	-	+
+	+	+	+	-	+	-

FLAIR, Fluid attenuated inversion recovery sequence; VDS, Venous distension sign.

A CSF study was carried out in five patients. It was not performed in two patients for fear of exacerbation of headache after lumbar puncture, and in one patient it was a dry tap. The CSF pressure was low in three patients and was normal in two patients. Two patients had elevation of CSF protein and mild lymphocytic pleocytosis, and one had a few RBCs in the CSF. All patients made complete recovery with conservative treatment, which included bed rest, good hydration, oral theophylline, and analgesics. None of them required any surgical intervention. The mean time to complete recovery was 25.6 days (range, 14-30 days). Follow-up was available on all patients. The mean follow-up period was 3.6 years (range, 1.2-7.7 years). All were asymptomatic at last follow-up except for an infrequent short vascular headache in two.

## Discussion

This is the largest series with sizeable number of patients with CHS with long-term follow-up from India. We encountered only eight cases of CHS over a 7-year period. It constituted 0.27% of total 2932 cases of patients with headache seen in our outpatient department over same study period. The prevalence of CHS was estimated to be around about 1 in 50,000 population in a study done in 1994.[7] Clinicians need to be vigilant toward this entity.

Several connective tissue disorders such as Marfanoid syndrome, Ehlers Danlos syndrome have been described in association with CHS.[8] None of the patients in this series had any evidence of such connective tissue disorders. The mean age of our patients was 40 years, which is similar to other studies.[1,2] The increased incidence of this condition among females seen in our study has been described in earlier studies.[1,9] This, however, has not been reported in few other series.[10] The underlying etiology for female preponderance is largely unknown. None of our patients had any immediate CSF leak prior to the diagnosis of CHS. However, in two patients there was history of spinal anesthesia for cesarean section more than a decade prior to presentation. They never had orthostatic headache till the current presentation. All our patients had orthostatic headache. The other symptoms such as neck pain may be secondary to traction on the pain-sensitive structures in the erect posture, secondary to hypovolemia.[1,2] The various ear symptoms such as tinnitus and fullness are postulated to be secondary to pressure gradient in the intra-labyrinthine compartment.[1,2] However, symptoms suggesting Parkinsonism and nonpostural headache described in other case reports were not observed in our patients.[1,2]

Three of the five patients who underwent CSF manometry had low pressure, whereas two had normal pressure. It is well documented that CSF pressure can be normal in CHS. In a series of 30 cases, 17.5% had normal CSF pressure.[11] This caveat that CSF pressure can be normal has led to the better nomenclature of condition as CHS rather than SIH. Typically, CSF cell count and protein levels are normal in CHS. CSF study showed mild pleocytosis (25%) in two patients and mild raise in protein in one patient. Chung *et al*.[1] noted raised protein and pleocytosis in 95% and 59%, respectively. The pleocytosis and elevated protein levels are attributed to subtle local inflammatory response.[1] This may also be secondary to compensatory meningeal vasodilatation. Although CSF pleocytosis and elevated proteins can occur in CHS, utmost care should be taken to exclude CSF infections and meningeal inflammation due to subarachnoid hemorrhage or other inflammatory diseases in such cases.

The MRI study of all the patients showed diffuse pachymeningeal gadolinium enhancement. The compensatory meningeal vasodilatation secondary to CSF hypovolemia is the proposed mechanism for this enhancement.[2,3] Some studies have shown that there can be no pachymeningeal enhancement in spite of CHS.[12] T2W and FLAIR sequence showed uniform hyperintensity of pachymeninges in all the cases in this series. This phenomenon is thought to be secondary to increased water content of meninges.[3] This finding can help to differentiate CHS from pachymeningitis, where meninges will be hypo intense in T2W sequence.[^-^3] All patients in our series had positive VDS on MRI and VDS disappeared with clinical resolution of symptoms in all three patients where follow-up imaging was done [Figure 2]. A recent study had shown that VDS is a characteristic sign in CHS and is the first sign to disappear after successful treatment.[13]

All patients in this series had improved on conservative treatment. None of them required epidural blood patch or other surgical procedures, which is in contrast to the observations in other series.[14] The various other treatment modalities described in refractory cases are epidural blood patches,[15,16] injection of hypertonic saline, and surgical repair of the dural tear.[7] The site of leak is detected by radioisotope study or CT myelogram. The cervical or thoracic dura are the usual site of leak.[1,2] We did not do any investigations to identify the site of leak as all patients improved with conservative management within a short period of time. According to the revised International Classification of Headache- 2 criteria,[17] clinical improvement after blood patch is a mandatory criterion for the diagnosis of CHS. Our observation that patients with CHS can recover without any blood patch contradicts this presumption. Long-term follow-up of our patients did not show any recurrence of similar headache. In another report,[4] 1 out of 13 patients had recurrence (7.6%) and 6 out of 13 had partial response to treatment on mean follow-up of 4.3 years. It appears that the nature history and course of CHS range from a benign self-limiting one to more refractory cases that require aggressive management. We could not ascertain any prognostic factors as all our patients made complete recovery with conservative treatment. The study by Schievink *et al*. showed that absence of pachymeningeal enhancement on cranial MRI in CHS is a bad prognostic sign.[18] The good outcome recorded in our series indicates that CHS can have a benign course and can undergo spontaneous remission with conservative treatment such as bed rest, hydration, and analgesics.

CHS constituted only 0.27% of the patients who presented with headache in our clinic. It is possible that CHS is rather under diagnosed. It is important to elicit the history of orthostatic provocation of the headache and investigate such cases with MRI with high-index of suspicion to arrive at correct diagnosis. Increased awareness of this rare condition among neurophysicians and family physicians is essential to diagnose this condition and to avoid unnecessary investigations. In majority of the CHS, it resolves spontaneously without any further recurrence.
